# Inhibition of Id proteins by a peptide aptamer induces cell-cycle arrest and apoptosis in ovarian cancer cells

**DOI:** 10.1038/sj.bjc.6605897

**Published:** 2010-09-14

**Authors:** D S Mern, J Hasskarl, B Burwinkel

**Affiliations:** 1 Helmholtz-University Group Molecular Epidemiology, German Cancer Research Center, Im Neuenheimer Feld 581, D-69120 Heidelberg, Germany; 2 Division of Hematology and Oncology, University of Freiburg Medical Center, Hugstetter Strasse 55, D-79106 Freiburg im Breisgau, Germany; 3 Division Molecular Biology of Breast Cancer, Department of Gynecology and Obstetrics, University of Heidelberg, Voss Strasse 9, D-69120 Heidelberg, Germany

**Keywords:** Id proteins, peptide aptamer, cell-cycle arrest, apoptosis, ovarian cancer

## Abstract

**Background:**

Inhibitors of DNA-binding proteins (Id1-4), lacking the basic DNA-binding domain, function as dominant inhibitors of cell-cycle regulators. Overexpression of Id proteins promotes cancer cell proliferation and resistance against apoptosis. Level of Id protein expression, especially of Id1, correlates with poor differentiation, enhanced malignant potential and more aggressive clinical behaviour of ovarian tumours. Although overexpression of Ids has been found and shown to correlate with poor clinical outcome, their inhibition at protein level has never been studied.

**Methods:**

A peptide aptamer, Id1/3-PA7, targeting Id1 and Id3, was isolated from a randomised combinatorial expression library using yeast and mammalian two-hybrid systems. Id1/3-PA7 was fused, expressed and purified with a cell-penetrating protein transduction domain.

**Results:**

Intracellular-delivered Id1/3-PA7 colocalised to Id1 and Id3. It induced cell-cycle arrest and apoptosis in ovarian cancer cells ES-2 and PA-1. It activated the E-box promoter and increased the expression level of cyclin-dependent kinase inhibitor (CDKN2A) in a dose-dependent manner that is paralleled by the cleavage of poly-ADP ribose polymerase. These effects were counteracted by ectopically overexpressed Id1 and Id3.

**Conclusion:**

Id1/3-PA7 could represent an exogenous anti-tumour agent that can significantly trigger cell-cycle arrest and apoptosis in ovarian cancer.

Inhibitors of DNA-binding or differentiation proteins (Id) belong to the helix–loop–helix (HLH) family of transcription factors. There are four members of the Id family, Id1 to Id4. Id proteins, lacking the basic DNA-binding domain, associate with members of the basic HLH (bHLH) transcription factors and form transcriptionally inactive heterodimers. Thus, they function as dominant-negative regulators of bHLH transcription factors (Benezra *et al*, 1990). Furthermore, Id proteins regulate cellular growth and senescence through direct sequestration of Ets and Rb proteins (Iavarone *et al*, 1994; Ohtani *et al*, 2001; Ruzinova and Benezra, 2003). Id proteins are essential for cell differentiation, proliferation, cell-cycle progression, migration and angiogenesis (Norton and Atherton, 1998; Israel *et al*, 1999; Norton, 2000). Although barely expressed in most normal tissues, deregulated expression of Id proteins has been demonstrated in a variety of human tumours (Lin *et al*, 2000; Schindl *et al*, 2001; Wang *et al*, 2004). Overexpression of Id proteins promotes cancer cell proliferation and resistance against apoptosis (Ouyang *et al*, 2002a, 2002b; Ling *et al*, 2003). Id overexpression in different tumours is significantly associated with poor clinical outcome (Schoppmann *et al*, 2003; Han *et al*, 2004). Level of Id protein expression, especially of Id1, correlates with poor differentiation, enhanced malignant potential and more aggressive clinical behaviour of epithelial ovarian tumours (Schindl *et al*, 2003). Recently, it has been shown that ectopic Id1 expression stimulates ovarian cancer cell proliferation, and this process is mediated through upregulation of epidermal growth factor receptor (EGFR), suggesting that Id1 might serve as an upstream regulator of the EGFR pathway in promoting ovarian cancer cell growth (Zhang *et al*, 2004). In various forms of tumours, including ovarian cancer, Id1 and Id3 are overexpressed extensively and in an overlapping pattern (Fong *et al*, 2000; Coppe *et al*, 2003). They have been shown to inactivate Ets1 and Ets2, which leads to the inhibition of CDKN2A expression and consequently allows phosphorylation of retinoblastoma protein (pRb) (Ohtani *et al*, 2001). Furthermore, DNA-binding motif (E-box)-mediated repression of the *CDKN2A* promoter by Id1 has been reported (Alani *et al*, 2001). A significant positive association has been reported between CDKN2A expression and clinical outcome for epithelial ovarian cancer patients (Kusume *et al*, 1999).

A peptide-conjugated Id1 antisense oligonucleotide homed to tumour endothelium inhibited tumour growth and metastasis in two different murine models (Henke *et al*, 2008). Mice lacking *Id1* and *Id3* genes (*Id1*^+/−^*Id3*^−/−^) are resistant to xenotransplanted tumour grafts and show defects in tumour neoangiogenesis (Lyden *et al*, 1999; de Candia *et al*, 2003). Therefore, Id1 and Id3 are considered as potentially therapeutic targets.

So far, a joint inhibition of Id1 and Id3 at protein level has hardly been studied. To develop a new strategy for the inhibition of Id1 and Id3, we isolated a novel peptide aptamer Id1/3-PA7 that specifically interacts with Id1 and Id3 (Mern *et al*, 2010). Peptide aptamers represent short peptides of random sequences. On the basis of their *in vivo* binding affinity to their target protein, they can be selected from randomised combinatorial expression libraries using yeast and mammalian two-hybrid systems (Fields and Song, 1989; Chien *et al*, 1991; Bartel *et al*, 1993). In this study, we investigated the role of the peptide aptamer Id1/3-PA7 on the progression of cell cycle and apoptosis in Id1- and Id3-overexpressing ovarian cancer cells ES-2 and PA-1. For its delivery into ovarian cancer cells, Id1/3-PA7 was fused with a cell-penetrating protein transduction domain (PTD) (Elliott and O'Hare, 1997; Phelan *et al*, 1998; Narita *et al*, 2001), expressed and purified from bacteria. We demonstrated that intracellular-delivered Id1/3-PA7 colocalised to Id1 and Id3 and promoted increased expression of the tumour suppressor CDKN2A in a dose-dependent manner. In addition, Id1/3-PA7 induced cleavage of the apoptosis indicator poly-ADP ribose polymerase (PARP). These effects were counteracted by ectopically overexpressed Id1 and Id3. Peptide aptamer Id1/3-PA7 significantly inhibited proliferation and induced apoptosis in ovarian cancer cells, with deregulated expression of Id1 and Id3. Therefore, we suggest that Id1/3-PA7 could represent an exogenous anti-tumour agent that could find applications in targeted therapy.

## Materials and methods

### Cell lines and cell culture

ES-2 and PA-1 cell lines were purchased in October 2007 from the American Type Culture Collection (ATCC, Manassas, VA, USA). PA-1 cell lines were grown in EMEM (ATCC), and ES-2 cell lines in McCoy's 5a (ATCC) supplemented with 10% fetal bovine serum (ATCC), penicillin (50 U ml^–1^), streptomycin (50 mg ml^–1^) and 2 mM glutamine (Sigma, Deisenhofen, Germany). All cell lines were tested and authenticated in February 2008 by the Genetic Core Facility at the German Cancer Research Center (Heidelberg, Germany) using the method Multiplex cell Contamination Test (McCT) (http://www.dkfz.de/gpcf/contamination-control.html).

### Transfections

For luciferase assays, ES-2 cells were transiently transfected using the calcium phosphate method (Chen and Okayama, 1987). For the analysis of CDKN2A expressions or PARP cleavage, ES-2 and PA-1 cells were transfected using FuGENE 6 Transfection Reagent (Roche Molecular Biochemicals, Indianapolis, IN, USA). Cells were selected with 1 mg ml^–1^ G418 (Sigma). Transfections were monitored by immunoblot detection of the expressed proteins.

### Bacterial expression and purification of PTD-fused peptide aptamers

A PTD, truncated VP22 ORF, was used for the intracellular delivery of the peptide aptamer (Elliott and O'Hare, 1997; Phelan *et al*, 1998; Narita *et al*, 2001). PTD was fused to the C-terminus of peptide aptamers or TrxA, which were inserted into vectors pCR T7/VP22-1-TOPO and pCR T7/VP22/NES-2-TOPO (Invitrogen, Carlsbad, CA, USA). Bacterial expression of the peptide aptamer was induced with isopropyl-1-thio-*β*-D-galactopyranoside (1 mM) for 5 h at room temperature. The peptide aptamers were furnished with 6xHis-tag to facilitate their purification by nickel chelate chromatography. The peptide aptamer was purified under native conditions using the ProBond Purification System (Invitrogen). The purity of the peptide aptamer was investigated by gel electrophoresis and Coomassie staining. The concentration was determined using the BCA Protein Assay Kit (Pierce, Rockford, IL, USA).

### Immunofluorescence and immunoblotting

For immunofluorescence analysis, cells were grown on coverslips, fixed and permeabilised in methanol for 20 min at −20°C and rehydrated with PBS. Before immunostaining with the indicated antibodies, cells were blocked in normal serum (1 : 10 in PBS containing 5% BSA). Nuclei were visualised by using Prolong Gold Antifade Reagent with DAPI (Invitrogen). Cells were analysed using a Carl Zeiss AxioVision 4 microscope equipped with a Carl Zeiss AxioCam digital camera and software version Carl Zeiss AxioVision Rel. 4.6.3 (Carl Zeiss Vision GmbH, Jena, Germany).

For immunoblot experiments, cell lysates prepared in EBC buffer (50 mM Tris–HCl, 120 mM NaCl, 1% (v/v) Nonidet P40, pH 8.0) supplemented with protease and phosphatase inhibitors were separated by SDS–PAGE and electrotransferred to polyvinylidene fluoride membranes (Millipore, Temecula, CA, USA). To detect antigen/antibody complexes, membranes were incubated with appropriate horseradish peroxidase-labelled secondary antibodies (Santa Cruz Biotechnology, Santa Cruz, CA, USA) and developed for enhanced chemiluminescence using the ECL WB Detection kit (Millipore).

Primary antibodies used were anti-Id1 (C-20), anti-Id3 (C-20) (Santa Cruz Biotechnology), anti-*α*-tubulin, anti-Trx (Sigma), anti-CDKN2A (Millipore) and anti-PARP (BD Biosciences, San Jose, CA, USA). Secondary antibodies used were goat anti-mouse or goat anti-rabbit Alexa Fluor 488, 660 and 680 for immunofluorescence experiments and horseradish peroxidase-conjugated goat anti-rabbit and goat anti-mouse (Santa Cruz Biotechnology) for immunoblotting.

### Coimmunoprecipitation

ES-2 and PA-1 cells were treated with peptide aptamer Id1/3-PA7 (5 *μ*g per 10^6^ cells). After 1.5 h, cells were lysed in RIPA buffer (Pierce) containing protease and phosphatase inhibitors. Protein samples were purified by nickel chelate chromatography under native conditions using the ProBond Purification System (Invitrogen) and analysed by western blotting using antibodies against Trx, Id1 and Id3.

### E-box promoter-reporter assays

pGL4.1-4Rtk-luc (E-box-dependent reporter construct), pcDNA3-E47, pCMV-Id1, pCMV-Id3, along with pCMV-LacZ as an internal standard, were used for transfection of ES-2 cells. After transfection, cells were treated by adding Id1/3-PA7 (5 *μ*g per 10^6^ cells) at 4 h intervals for 48 h. Cells were lysed in Triton buffer (1% Triton X-100, 25 mM glycylglycin (pH 7.8), 15 mM MgSO_4_, 4 mM EGTA and 1 mM DTT) for 10 min on ice. The lysates were clarified by centrifugation for 10 min at 16 000 **g**. Lysates (10 *μ*l) were measured using a luminometer (Berthold, Vista, CA, USA) by injecting luciferin reagent (25 mM glycylglycin (pH 7.8), 5 mM ATP (pH 7.8) and 330 mM beetle luciferin). The samples were normalised for *β*-galactosidase activity, which was measured after incubating 3 *μ*l lysate with 33 *μ*l reaction buffer (100 mM Na_2_HPO_4_, 1 mM MgCl_2_ and 1 × Galacton (Tropix, Bedford, MA, USA)) for 30 min in the dark. Galactosidase activity was measured in a luminometer by injection of amplifier (10% Emerald (Tropix), 0.2 M NaOH).

### RNA analysis

Cells were treated with different doses of Id1/3-PA7 (1–7.5 *μ*g per 10^6^ cells) at 4 h intervals for 48 h. Total RNA was purified using an RNeasy Plus Mini Kit (Qiagen, Hilden, Germany) and inspected by agarose gel electrophoresis. cDNAs were synthesised using TaqMan Reverse Transcription Reagents (Applied Biosystems, Foster City, CA, USA). The mRNAs of CDKN2A, CDKN1A, CDKN1B and *β*-actin as internal standard were measured by real-time PCR with TaqMan gene expression assays (Applied Biosystems), using the LightCycler 480 Real-Time PCR System and LightCycler 480 Probes Master (Roche Applied Science, Mannheim, Germany) under the following conditions: 15 min at 95°C activation, 40 cycles of 15 s at 95°C, followed by 30 s at 55°C and 1 s at 72°C, melting for 1 s at 55°C and cooling for 1 min at 40°C. For the relative quantification of mRNA levels, three independent amplifications were performed for each probe, with triplicate samples.

### Flow cytometry cell-cycle analysis

Cells were treated with Id1/3-PA7 (5 *μ*g per 10^6^ cells) at 4 h intervals for 48 h. For quantitative cell-cycle analysis, the BD Pharmingen FITC bromodeoxyuridine (BrdU) Flow Kit (BD Biosciences) was used. Prolonged cell exposure (6 h) to BrdU allowed the identification and analysis of actively cycling, as opposed to non-cycling, cell fractions. Cell-incorporated BrdU (with FITC anti-BrdU) and total DNA content (with 7-AAD) in cells were measured using the BD FACSCalibur Flow Cytometer (BD Biosciences).

## Results

### Peptide aptamer Id1/3-PA7 colocalises to Id1 and Id3

**Figure 1:**
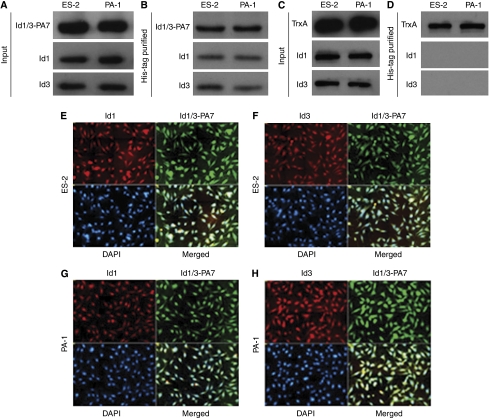
Colocalisation of the intracellular-delivered peptide aptamer Id1/3-PA7 to Id1 and Id3. (**A**, **B**) His-tag coimmunoprecipitation of Id1/3-PA7 with Id1 and Id3 in ovarian cancer cells ES-2 and PA-1. (**C**, **D**) His-tag coimmunoprecipitation of TrxA as negative control. (**E**–**H**) Coimmunofluorescence staining of Id1/3-PA7 with Id1 and Id3 in ES-2 and PA-1 cells. DAPI, 4,6-diamidino-2-phenylindole.

Using yeast and mammalian two-hybrid systems, we isolated from the randomised expression library a constrained peptide aptamer Id1/3-PA7 that interacts specifically with Id1 and Id3 (Mern *et al*, 2010). Here, we used Id1/3-PA7 to analyse its functional interference in Id1- and Id3-overexpressing ovarian cancer cells ES-2 and PA-1. For its delivery into ovarian cancer cells, Id1/3-PA7 was fused with cell-penetrating PTD (Elliott and O'Hare, 1997; Phelan *et al*, 1998; Narita *et al*, 2001), expressed in bacteria and purified under native conditions. Ovarian cancer cells ES-2 and PA-1 were treated with Id1/3-PA7 (5 *μ*g per 10^6^ cells). At 1.5 h after treatment, the internalisation of the peptide aptamer into cells and its colocalisation with Id1 and Id3 were verified by His-tag coimmunoprecipitation (Figure 1A and B). Cells treated with TrxA (5 *μ*g per 10^6^ cells) were used as negative control in coimmunoprecipitation (Figure 1C and D). In addition, the colocalisation was verified by coimmunofluorescence staining (Figure 1E–H).

### Functional interference of Id1/3-PA7 with Id1 and Id3

**Figure 2:**
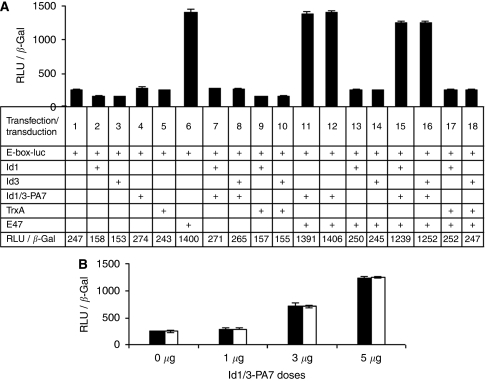
(**A**) Peptide aptamer Id1/3-PA7 restores the E-box promoter activity. The transcription factor E47 upregulated the E-box promoter-dependent reporter gene (6), Id1 and Id3 reduced E47-dependent activation (13, 14) and addition of peptide aptamer Id1/3-PA7 restored E47-dependent activation (15, 16). The control with TrxA showed no effect on E-box promoter activity. (**B**) With Id1 and Id3, reduced E47-dependent activation is restored by the addition of Id1/3-PA7 in a dose-dependent manner (RLU/*β*-Gal: 250, 290, 720 and 1252 units respectively). Bars: black, Id1; white, Id3. Results are represented as mean values of three independent experiments with s.d. (*P*⩽0.05).

E-proteins activate transcription by binding to promoter E-boxes. Formation of heterodimers between Id protein and E-protein prevents E-proteins from forming DNA-binding transcriptionally active complexes. To analyse the potency of Id1/3-PA7 with respect to Id1 and Id3 inhibition and restoration of E-box promoter activity, we used the E-box-dependent reporter gene *4Rtk-luc*, which contains four tandem E-boxes from the MCK enhancer upstream of the thymidine kinase basal promoter (Weintraub *et al*, 1990). ES-2 cells were transfected with 4Rtk-luc, pcDNA3-E47, pCMV-Id1, pCMV-Id3 and pCMV-LacZ as an internal standard. After transfection, cells were treated with different doses of Id1/3-PA7 (1, 3 and 5 *μ*g per 10^6^ cells, half-life 2.5 h) at 4 h intervals for 48 h. The reporter gene was upregulated in the presence of the E-protein, E47. E47-dependent activation was reduced in the presence of Id1 or Id3, and, by addition of Id1/3-PA7, the activation was restored again (Figure 2A). Id1/3-PA7 restored E47-dependent activation in a dose-dependent manner (Figure 2B and Supplementary data 1). Treatment of cells with different doses of TrxA ((1, 3 and 5 *μ*g per 10^6^ cells) had no effect on E-box promoter activity (Figure 2A and Supplementary data 1).

### Id1/3-PA7 enhances the expression level of CDKN2A

**Figure 3:**
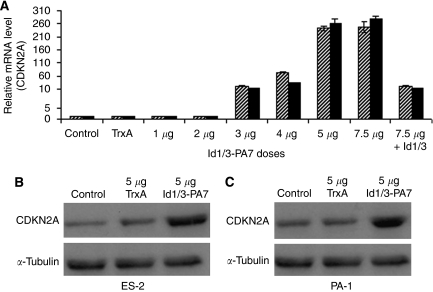
Intracellular-delivered Id1/3-PA7 increases the expression level of CDKN2A. (**A**) Results of quantitative real-time PCR. Intracellular-delivered Id1/3-PA7 caused a dose-dependent increase in the relative mRNA level of CDKN2A compared with that in untreated or TrxA-treated (5 *μ*g per 10^6^ cells) cells. The increased expression level of CDKN2A was counteracted by ectopically overexpressed Id1 and Id3. Bars: hatched, ES-2 cells; black, PA-1 cells. Results are represented as mean values of three independent experiments with s.d. (*P*⩽0.05). (**B**, **C**) Western blotting showed increased CDKN2A protein levels in Id1/3-PA7-treated (5 *μ*g per 10^6^ cells) ES-2 and PA-1 cells, compared with that in untreated or TrxA-treated (5 *μ*g per 10^6^ cells) cells.

E-box promoter-mediated repression of CDKN2A, CDKN1A and CDKN1B expression by Ids has been reported (Prabhu *et al*, 1997; Alani *et al*, 2001; Perk *et al*, 2005). Furthermore, it has been shown that Id1 and Id3 inactivate Ets1 and Ets2 and inhibit the transcriptional expression of CDKN2A (Ohtani *et al*, 2001). Therefore, we analysed the effect of Id1/3-PA7 on the expression level of CDKN2A, CDKN1A and CDKN1B, using real-time PCR and western blotting. Ovarian cancer cells ES-2 and PA-1 were treated with different doses of Id1/3-PA7 (1–7.5 *μ*g per 10^6^ cells) at 4 h intervals for 48 h. Intracellular delivery of Id1/3-PA7 caused a dose-dependent increase in the mRNA and protein levels of CDKN2A (Figure 3A and B). Untreated cells or cells treated with TrxA (5 *μ*g per 10^6^ cells) did not show any change in CDKN2A expression (Figure 3A and B). This increased expression level of CDKN2A is Id1 and Id3 specific, being counteracted by ectopically overexpressed Id1 and Id3 (Figure 3A). The expression levels of CDKN1A and CDKN1B were moderately increased after treatment with only high doses of Id1/3-PA7 (5 and 7.5 *μ*g per 10^6^ cells) (Supplementary data 2). However, these moderately increased expression levels of mRNA could not be confirmed at protein level. The treatment of PA-1 and ES-2 cells with lower doses of Id1/3-PA7 (1–4 *μ*g per 10^6^ cells) did not change the expression levels of CDKN1A and CDKN1B (Supplementary data 2).

### Id1/3-PA7 induces cell-cycle arrest and apoptosis in ovarian cancer cells

**Figure 4:**
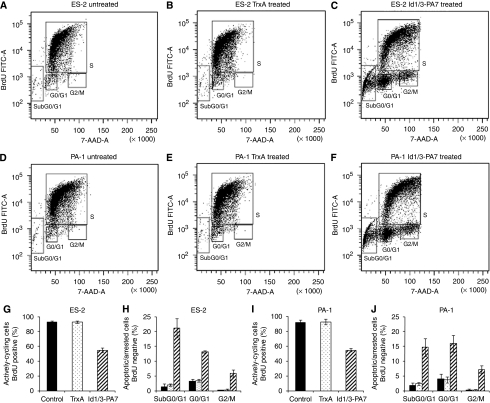
Id1/3-PA7 induces cycle arrest and apoptosis in ovarian cancer cells. (**A**–**J)** Quantification of cell-incorporated bromodeoxyuridine (BrdU) (fluorescein isothiocyanate (FITC) anti-BrdU) and total DNA content (7-AAD) in untreated and TrxA-treated (5 *μ*g per 10^6^ cells) or Id1/3-PA7-treated (5 *μ*g per 10^6^ cells) ES-2 cells (**A**, **C**) and PA-1 cells (**D**–**F**). Flow cytometric analysis of untreated cells *vs* Id1/3-PA7-treated cells showed S-phase reduction of actively cycling ES-2 cells (**G**) and PA-1 cells (**I**), increasing apoptotic cells in sub-G0/G1- and G2/M-resided ES-2 cells (**H**) and PA-1 cells (**J**). There were no significant differences between untreated and TrxA-treated cells (**A**–**J**). Bars: black, untreated; white dotted, TrxA treated; hatched, Id1/3-PA7 treated. Results are represented as mean values of three independent experiments with s.d. (*P*⩽0.05).

Tumour suppressor CDKN2A inhibits the cyclin-dependent kinases (Cdk4 and Cdk6) that initiate the phosphorylation of the pRb (Ruas and Peters, 1998; Sherr and Roberts, 1999). Therefore, CDKN2A has the capacity to arrest cells in the G1 phase of the cell cycle. Applying immunofluorescent staining of incorporated BrdU, an analogue of the DNA precursor thymidine, and flow cytometric analysis, we quantified individual cells that have incorporated BrdU into newly synthesised DNA as cells entering and progressing through the S (DNA synthesis) phase of the cell cycle. ES-2 and PA-1 cells were treated with TrxA or Id1/3-PA7 (5 *μ*g per 10^6^ cells) at 4 h intervals for 48 h. For the identification and analysis of actively cycling, as opposed to non-cycling, cell fractions, we exposed untreated and TrxA- or Id1/3-PA7-treated cells to BrdU for 6 h. The cell-cycle positions and active DNA synthetic activities of cells were determined by analysing the correlated expression of total DNA and incorporated BrdU levels as shown by the region gates applied to the 7-AAD *vs* BrdU dot plot (Figure 4A–F). Flow cytometric analysis of untreated cells *vs* Id1/3-PA7-treated cells demonstrated that the anti-proliferative and apoptotic effects of Id1/3-PA7 reduced the number of actively cycling cells in S from 93.1 to 54.8% for ES-2 cells (Figure 4G) and from 91.4 to 54.6% for PA-1 cells (Figure 4I). The number of apoptotic cells in sub-G0/G1- and in G0/G1- or G2/M-resided cells increased from 1.4 to 21.1%, from 3.2 to 13.2% and from 0.2 to 5.9%, respectively, for ES-2 cells (Figure 4H), and from 1.7 to 14,6%, from 3.9 to 16% and from 0.3 to 7.2%, respectively, for PA-1 cells (Figure 4J). There were no significant differences between untreated and TrxA-treated cells (Figure 4A–J).

### Apoptotic PARP cleavage in response to Id1/3-PA7

**Figure 5:**
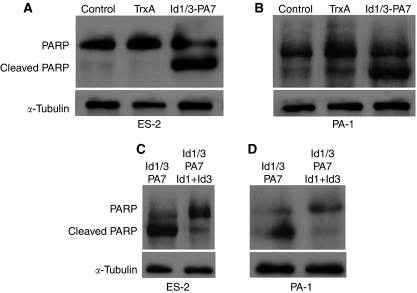
Apoptotic poly-ADP ribose polymerase (PARP) cleavage induced by Id1/3-PA7. (**A**, **B**) Untreated and TrxA- or Id1/3-PA7-treated (5 *μ*g per 10^6^ cells) ES-2 and PA-1 cells were analysed for PARP cleavage by western blotting. PARP cleavage was detected in cell extracts of Id1/3-PA7-treated cells. (**C**, **D**) In Id1/3-PA7-treated cells, PARP cleavage was counteracted by ectopically overexpressed Id1 and Id3.

It has been reported that wild-type CDKN2A expression from an adenovirus vector (Adv/p16) in non-small-cell lung cancer cell line A549, which carries the wild-type p53 gene, results in activation of caspase-3, accompanied by the cleavage of its substrate PARP (Koh *et al*, 2002). Furthermore, it has been shown that ectopically overexpressed Id1 is able to suppress PARP cleavage in response to different anticancer drugs, which leads to increased apoptosis rates and increased cleaved PARP in different cancer cell lines (Zhang *et al*, 2007). Therefore, Id1/3-PA7-treated ES-2 and PA-1 cells were analysed for PARP cleavage compared with untreated or TrxA-treated cells. We detected PARP cleavage by western blotting in cell extracts of Id1/3-PA7-treated cells using anti-PARP antibody, which recognises uncleaved PARP (113 kDa) and cleaved PARP (85 kDa) (Figure 5A and B). PARP cleavage was counteracted in Id1/3-PA7-treated cells by ectopically overexpressed Id1 and Id3 (Figure 5C and D).

## Discussion

Id1 and Id3 are considered as potentially versatile therapeutic targets. In various forms of tumours, Id1 and Id3 are overexpressed extensively and in an overlapping pattern (Fong *et al*, 2000; Coppe *et al*, 2003). The level of Id protein expression correlates with poor differentiation, enhanced malignant potential, aggressive clinical behaviour of different tumours, including epithelial ovarian tumours, and is a strong predicator of shorter survival (Schindl *et al*, 2003). Depending on the tumour art and stage, partial loss of Id functions is sufficient to have a therapeutic effect, as shown in *Id1*^+/−^*Id3*^−/−^ knockout mice (Lyden *et al*, 1999; de Candia *et al*, 2003). It has also been shown that Id1 and Id3 overexpression correlates with loss of CDKN2A expression in different tumours (Polsky *et al*, 2001; Lee *et al*, 2003), suggesting that in some settings Id1/3-induced cell proliferation is mediated through the inactivation of the CDKN2A-pRb pathway. Therefore, we investigated the biological effect of the peptide aptamer Id1/3-PA7 in Id1/Id3-overexpressing and CDKN2A-positive ovarian cancer cells, ES-2 and PA-1. In this paper, we demonstrated that inhibition of Id1 and Id3 by the peptide aptamer Id1/3-PA7 induced cell-cycle arrest and apoptosis. It significantly increased the endogenous expression level of CDKN2A in a dose-dependent manner, which is paralleled by the cleavage of PARP. In contrast to this, the expression levels of CDKN1A and CDKN1B were moderately increased after treatment with only high doses of Id1/3-PA7 (5 and 7.5 *μ*g per 10^6^ cells) (Supplementary data 2). However, these moderately increased expression levels of mRNA could not be confirmed at protein level. The treatment of PA-1 and ES-2 cells with lower doses of Id1/3-PA7 (1–4 *μ*g per 10^6^ cells) has not changed the expression levels of CDKN1A and CDKN1B (Supplementary data 2). This could indicate that in PA-1 and ES-2 ovarian cancer cells, Id1/3-PA7 might preferentially inhibit the heterodimerisation of Id1 and Id3 with ETS proteins, which are the positive regulators of CDKN2A. Previously, we have shown that Id1/3-PA7 regulates the expression of CDKN1A and CDKN1B in a dose-dependent manner in breast cancer cells MCF-7 and MDA-MB-231, which contain the homozygous deletions of the gene *CDKN2A* (Kamb *et al*, 1994). These results together suggest that Id1 and Id3, depending on the tumour type, might differentially regulate the expression of tumour suppressor genes *CDKN2A*, *CDKN1A* and *CDKN1B*.

Besides the CDKN/pRb pathways, Id1/3-PA7 could interestingly be used to analyse EGFR pathway-dependent functions, as Id proteins, especially Id1, induce upregulation of EGFR in different tumour cells, very frequently in androgen-independent prostate cancer cells. Thus, Id1/3-PA7 could have effects on androgen-independent proliferation of prostate cancer cells. Overexpression of Id1 is associated with progression of prostate cancer and Id1-induced androgen-independent prostate cancer cell growth is correlated with upregulation of EGFR (Ouyang *et al*, 2002a, 2002b; Ling *et al*, 2004).

In our previous work (Mern *et al*, 2010), we demonstrated using a mammalian two-hybrid system that Id1/3-PA7 interacts with Id1 and Id3 but does not bind to Id2, Id3 and the interacting partners of Id proteins, such as E47, MyoD, ETS1, ETS2 and S5A. Furthermore, we have shown that the biological effects of Id1/3-PA7, in analysed breast and ovarian cancer cells, were counteracted by forced expression of Id1 and Id3. These results indicate that the biological effects of Id1/3-PA7 are based on the functional blocking of Id1 and Id3. We hope that future *in vivo* application of the peptide aptamer Id1/3-PA7 in tumour-bearing Id1/3 transgenic mice and in *Id1*^+/−^*Id3*^−/−^ knockout mice will give rise to more information regarding its *in vivo* stability, functional specificity and potential toxicity.

Overexpression of Id proteins, especially of Id1, has been found to be correlated with the progression of different types of solid tumours (Perk *et al*, 2005; Ling *et al*, 2006). Their low postnatal expression and their roles in tumourigenesis and tumour neoangiogenesis mark them as attractive targets for anticancer therapy (Perk *et al*, 2005). Therefore, we suggest that Id1/3-PA7, as inhibitor of Id1 and Id3, could have the potential to be used as a new tool for targeted tumour therapy.
